# Socioeconomic inequalities in the non-use of dental care in Europe

**DOI:** 10.1186/1475-9276-13-7

**Published:** 2014-01-29

**Authors:** Anastase Tchicaya, Nathalie Lorentz

**Affiliations:** 1 CEPS/INSTEAD, 3 Avenue de la Fonte, L-4364, Esch-sur-Alzette, Luxembourg

**Keywords:** Oral health, Dental care, Non-use of dental care, Socioeconomic inequality, Socioeconomic determinants, Human development index, Density of dentists, Multilevel analysis, Europe

## Abstract

**Introduction:**

Oral health is an important component of people’s general health status. Many studies have shown that socioeconomic status is an important determinant of access to health services. In the present study, we explored the inequality and socioeconomic factors associated with people’s non-use of dental care across Europe.

**Methods:**

We obtained data from the European Union Statistics on Income and Living Conditions survey conducted by Eurostat in 2007. These cross-sectional data were collected from people aged 16 years and older in 24 European countries, except those living in long-term care facilities. The variable of interest was the prevalence of non-use of dental care while needed. We used the direct method of standardisation by age and sex to eliminate confounders in the data. Socioeconomic inequalities in the non-use of dental care were measured through differences in prevalence, the relative concentration index (RCI), and the relative index of inequality (RII). We compared the results among countries and conducted standard and multilevel logistic regression analyses to examine the socioeconomic factors associated with the non-use of dental care while needed.

**Results:**

The results revealed significant socio-economic inequalities in the non-use of dental care across Europe, the magnitudes of which depended on the measure of inequality used. For example, inequalities in the prevalence of non-use among education levels according to the RCI ranged from 0.005 (in the United Kingdom) to −0.271 (Denmark) for men and from −0.009 (Poland) to 0.176 (Spain) for women, whereas the RII results ranged from 1.21 (Poland) to 11.50 (Slovakia) for men and from 1.62 (Poland) to 4.70 (Belgium) for women. Furthermore, the level-2 variance (random effects) was significantly different from zero, indicating the presence of heterogeneity in the probability of the non-use of needed dental care at the country level.

**Conclusion:**

Overall, our study revealed considerable socioeconomic inequalities in the non-use of dental care at both the individual (intra-country) and collective (inter-country) levels. Therefore, to be most effective, policies to reduce this social inequality across Europe should address both levels.

## Introduction

Oral health is an important component of overall health; thus, poor oral health can have negative implications for people’s general health status. The main objective of any health care system is to provide the population with equal access to health care, regardless of socioeconomic status or geographic location. However, in practice, the diversity of health needs and individual characteristics, as well as the heterogeneity of clinical practice, make it difficult to achieve this goal [1].

Prior research has shown that socioeconomic status is an important determinant of health-service utilization [2-8]; specifically, individuals who belong to higher socioeconomic groups generally use a wider range of health services than those in lower socioeconomic groups [6,9,10]. Results of the European Community Household Panel Wave 3 [11] revealed an inequality favouring the richest people across all European countries when the concentration indices associated with dental visits were found to be positive and significantly different from zero. In contrast, people from lower socioeconomic groups were more likely than others to forgo dental care. However, findings varied according to the country and the type of health care resources used.

Access to healthcare in general remains a major issue in many countries, even those with a universal health-care insurance scheme [8,12-15]. Several recent studies [8,15,16] focused on the use or coverage of dental services across a number of countries: the results have highlighted the improvement of dental care as an international priority. Such improvement can be facilitated by addressing the inequities in dental care both within and across countries.

The behavioural model of access to healthcare, developed mainly by Andersen [17], provides an appropriate conceptual framework for the analysis of the various socioeconomic determinants of the non-use of healthcare in Europe. According to this model, the use of health services is a function of individual predispositions (i.e. demographic and social characteristics), enabling factors such as the characteristics of the country’s health system (density of dentists –representing the number of dentists per 10,000 people, insurance coverage), various macroeconomic environmental factors (e.g. the Human Development Index (HDI)), and needs factors. In other words, characteristics on both the individual and collective levels influence how people perceive the need for, demand, and actual use of the health care services [17-19].

This study addresses the problem of dental-care access in particular by focusing on the renunciation, or non-use, of dental care in Europe by analysing data from a large group of European countries. Specifically, we examined the role of inequality and socioeconomic determinants in the non-use of dental care because this topic has received relatively little research attention; previous studies have focused more on people’s actual use of health services (including dental care) than on their ability to access such services [2-4]. Our main purpose in conducting this comparative analysis was to identify (i) the extent of the inequality in the non-use of dental care in Europe and (ii) the socioeconomic determinants that contribute to this inequality.

## Methods

### Sources and data

The data for this study were extracted from the European Union Statistics on Income and Living Conditions (EU-SILC) for 2007, a survey conducted across institutions in different European countries under EU regulation with the coordination of Eurostat. The cross-sectional data pool of the EU-SILC allows researchers to track changes in the income and living conditions of households for use in social protection policies [20]. Furthermore, the EU-SILC contains items addressing the non-use of dental care, including the main reasons for the lack of use. We used data from 24 European countries for individuals aged 16 and older. People living in institutions, that is, hospitals, retirement homes, or long-term care facilities, were not surveyed as part of the EU-SILC. The sample size from all countries was 389,405 persons aged 16 and older; sample sizes for each country ranged from 6,567 (Ireland) to 44,629 (Italy; see Table 1).

**Table 1:** Sample size of the 2007 SILC survey, by gender and country, EU-survey Statistics on Income and Living Conditions (SILC), 2007

	**Males**	**Females**	**Total sample size**
Austria	6,332	7,059	13,391
Belgium	5,972	6,350	12,322
Cyprus	4,040	4,430	8,470
Czech Republic	9,094	10,290	19,384
Denmark	5,709	5,901	11,610
Estonia	5,524	6,447	11,971
Spain	13,643	15,013	28,656
Finland	11,082	10,691	21,773
France	9,724	10,633	20,357
Greece	5,932	6,414	12,346
Hungary	8,357	10,133	18,490
Ireland	5,142	5,750	10,892
Iceland	3,320	3,247	6,567
Italy	21,264	23,365	44,629
Lithuania	5,000	5,913	10,913
Luxembourg	3,942	3,971	7,913
Latvia	3,924	5,346	9,270
The Netherlands	9,626	9,997	19,623
Norway	5,892	5,810	11,702
Poland	16,507	18,381	34,888
Portugal	4,665	5,282	9,947
Sweden	7,069	7,135	14,204
Slovak Republic	5,859	6,744	12,603
United Kingdom	8,340	9,144	17,484
All countries	185,959	203,446	389,405

To explore the effects of macroeconomic factors on the non-use of dental care, data from the United Nations Programme for Development (UNDP) were included with the main pool of EU-SILC data to be analysed. This included in particular the Human Development Index (HDI) [21] of each of the 24 countries, along with the proportion of dentists per 10,000 people (i.e. the density of dentists; see Table 2) as determined by the World Health Organization (WHO) [22]. Finally, we added a measure of the degree of dental-care insurance coverage in each country using data from the OECD Survey on Health System Characteristics 2008–2009 and OECD estimates [23].

**Table 2:** Classification of countries by the human development index, density of dentists and coverage of dental care

**HDI group**	Countries (HDI*)	Density of dentists (ratio of dentists per 10 000 inhabitants)**	**Countries (density of dentists)**	Coverage of dental care (CODCARE) (%)***	**Countries (CODCARE)**
HDI group 1	Estonia (0.860)	Group 1	Austria (5)	CODCARE 0	Czech Republic (1–50)
(HDI < =0.874)	Hungary (0.874)	Density <6	Poland (3)	(0-50%)	Denmark (1–50)
	Lithuania (0.862)		Slovak republic (5)		France (1–50)
	Latvia (0.855)		Hungary (5)		Greece (1–50)
	Poland (0.870)		Spain (5)		Hungary (1–50)
	Slovak Republic (0.863)		The Netherlands (5)		Ireland (0)
					Italy (1–50)
					The Netherlands (1–50)
					Norway (0)
					Portugal (1–50)
					Sweden (1–50)
HDI group 2	Austria (0.948)	Group 2	Portugal (6)	CODCARE 1	Austria (100)
(0.874 – 0.952)	Belgium (0.946)	density: 6-7	Italy (6)	(51-100%)	Belgium (76–99)
	Cyprus (0.903)		Ireland (6)		Spain (100)
	Czech Republic (0.891)		Latvia (7)		Finland (76–99)
	Denmark (0.949)		Lithuania (7)		Iceland (76–99)
	Spain (0.949)		Czech Republic (7)		Luxembourg (51–75)
	Greece (0.926)		France (7)		Poland (100)
	Italy (0.941)				Slovak Republic (51–75)
	Luxembourg (0.944)				United Kingdom (76–99)
	Portugal (0.897)				
	United Kingdom (0.946)				
HDI group 3	Finland (0.952)	Group 3	Belgium (8)		
(HDI > =0.952)	France (0.952)	Density >7	Luxembourg (8)		
	Ireland (0.959)		Denmark (8)		
	Iceland (0.968)		Sweden (8)		
	The Netherlands (0.953)		Estonia (9)		
	Norway (0.968)		Cyprus (9)		
	Sweden (0.956)		Finland (9)		
			Norway (9)		
			United Kingdom (10)		
			Iceland (10)		
			Greece (12)		

(*)United Nations Development Programme\Human Development Report 2007/2008.

(**)WHO\World Health Statistics 2009.

(***)OECD Survey on health system characteristics 2008–2009 and OECD estimates.

### Prevalence of the non-use of dental care

The prevalence of the non-use of dental care represents the outcome variable, a binary value defined by ‘yes’ or ‘no’ responses to whether individuals had experienced an ‘unmet need for dental examination or treatment during the last 12 months’. A ‘yes’ response indicated that there had been at least one occasion when the person needed a dental examination or treatment but did not receive it. Thus, the outcome variable was defined as follows: ‘non-use’ = 1, and ‘use or no dental problems’ = 0. The second variable concerns the prevalence of the non-use of dental care for financial reasons among people who renounced care based on the question, ‘[the] reason for unmet need for dental examination or treatment’, with ‘could not afford to (too expensive)’ = 1 and all other reasons = 0. The total prevalence of the non-use of dental care and the non-use of dental care for financial reasons represents the total prevalence of the unmet need for dental care over the previous 12 months.

### Socioeconomic and demographic variables

The main individual-level variables (from the EU-SILC) were education level, marital status, age, sex, employment status, and standard of living; these are described in more detail, as follows.

Education level, defined as the highest level of education successfully obtained according to the International Standard Classification of Education (ISCED) adopted by UNESCO [24], is divided into three levels: primary education (i.e. the first stage of basic education), secondary education (i.e. lower secondary education or the second stage of basic education, graduate of secondary education, and post-secondary education that is not higher education), and higher education (first cycle of higher education, the second stage of tertiary education).

Marital status has four categories in the EU-SILC: never married, married, divorced/separated, and widowed. Age was used as a continuous variable. Professional status was also divided into four categories: employee or self-employed, student, retired or pre-retired, and other.

Finally, standard of living refers to household disposable income, divided into quartiles. Equivalent income is calculated as disposable income divided by the equivalent household number of consumption units. Equivalent income is defined here by a commonly used quartile (or quintile) structure: the first quartile corresponds to individuals in the 25% of households with the lowest standard of living, and the fourth corresponds to those in the 25% of households with the highest standard of living.

### Macroeconomic variables

The HDI is a composite index developed by the UNDP to classify countries according to their level of human development. HDI values correspond to the arithmetic mean of three main socioeconomic indicators: GDP per capita, life expectancy at birth, and school enrolment. HDI scores were divided into three groups for its use as a categorical variable: group 1 (HDI ≤ 0.874), group 2 (HDI: 0.875–0.951), and group 3 (HDI ≥ 0.952) (see Table 1). The HDI values for the cut-off point were calculated using the quartiles. We combined the second and third quartiles into one group because the number of observed countries was small. Thus, we retained three groups. Note that all 24 countries in our study belong to the group of countries with a high index of human development [21].

The density of dentists (the ratio of dentists per 10,000 inhabitants) was defined as a categorical variable split into three categories (density < 6, density = 6–7, and density > 7) to serve as an indicator of the availability of dental care (see Table 2).

Finally, insurance coverage for dental care was used as an indicator of the degree of support for basic dental-care costs by the healthcare system in each country. This variable, originally defined as a percentage of costs covered (share of costs covered by basic primary health insurance) [23], was dichotomized as follows: coverage >50% of basic dental care costs = 1; coverage ≤ 50% = 0. Data were only available for 20 countries for this variable (see Table 2).

### Statistical analysis

Two types of statistical analyses were conducted: (i) comparisons between summary measures of socioeconomic inequalities using education level as an indicator of socioeconomic position and (ii) logistic regression analyses for assessing the association between the prevalence of the non-use of dental care and individual and contextual characteristics.

(i) The comparisons between summary measures of socioeconomic inequalities allowed us to make comparisons between countries, thus increasing the diversity of situations. Then, to limit the effect of confounding variables, the data were standardised by age and sex using the direct method [25], with the European standard population as the reference population [26]. The main summary measures of socioeconomic inequalities in the non-use of dental care were the ‘difference in prevalence’, ‘relative concentration index’ (RCI), and ‘relative index of inequality’ (RII) [27-31]. We specifically assessed the non-use of dental care in terms of education level. Difference in prevalence refers to the difference in the non-use between people with primary education and those with higher education.

The RCI refers to the relative concentration distribution of the non-use of dental care according to education level. A negative value for this index indicates a concentration inequality (in terms of non-use or unmet dental-care need) among people with lower education levels.

We used the formula for calculating the RCI to the grouped data proposed by Kakwani et al. [27]:

$$\text{RCI}=\frac{2}{\mu }\left[\sum_{j=1}^{j}P_{j}\mu_{j}R_{j}\right]-1$$

Where, in our case:

*P*_*j*_ is the group’s population share;

***μ***_*j*_ is the group’s mean prevalence of the non-use of dental care (or unmet dental-care need);

*R*_*j*_ is the relative rank of the J^th^ socioeconomic group, which is defined as follows:

$$R_{j}=\sum_{j=1}^{J}P_{j}-\frac{1}{2}P_{j}$$

Where *P*_***γ***_ is the cumulative share of the population up to and including group j, and *P*_*j*_ is the share of the population in group j.

The possible values of the RCI for a binary outcome are limited by the mean of the distribution. Thus, for ***μ*** ≠ 0, the RCI had a minimum [***μ****–*1 + 1/*n*] and maximum [1*–****μ*** + 1/*n*]; *n* is the sample size [32].

Finally, the RII takes into account the relative importance of each education group. Its value is calculated by comparing the extreme groups [28]. Values greater than 1 suggest a large inequality; here, that inequality concerns people who have less education.

In this study, and similar to previous research [29,30], we placed more emphasis on the RII because it is a simpler way to compare the magnitude of the association between one measure of socioeconomic position (in our case, education level) and the outcome (here, the prevalence of forgoing dental care) in different populations. In the EU-SILC, the factors used in calculating the weighted data differ across countries. In the current study, our measures were calculated with these weighted data and adjusted for age.

(ii) We used two types of logistic regression models to analyse the socioeconomic factors associated with the non-use of dental care: (1) traditional (i.e. one level) and (2) multilevel.

(1) For the traditional logistic regression, five logistic regression models were applied. The first model assessed the probability of the non-use of dental care solely in terms of the individual data. In the second model, we added the density of dentists within each country. In the third and fourth, we added the HDI and the degree of dental-care insurance coverage in each country, respectively. The final model incorporated the three contextual variables.

(2) For the multilevel logistic regression models, we used a multilevel analysis with two levels. Six multilevel logistic regression models were performed in order to measure the individual- and country-level socioeconomic inequalities in the non-use of dental care. The multilevel logistic regression has been used in several previous epidemiological studies [33-40].

Assuming that the probability of the non-use of dental care may be statistically dependent on both the variance of individual characteristics (level 1) and the contextual characteristics (level 2), we considered a multilevel logistic regression analysis based on a logit link function and a function of binomial distribution [41-45]. Our multilevel logistic model with K level-1 explanatory variables x_1_, x_2_ …, x_k_ and L level-2 variables explanatory variables z_1_, z_2_, …, z_l_ has the following form:

$$\begin{array}{l} \text{logit}\left(Y_{\text{ij}}\right)=\text{logit}\left(\frac{P_{\text{ij}}}{1-P_{\text{ij}}}\right)=a_{00}+\sum_{k=1}^{K}\beta_{k0}x_{\text{kij}}+\sum_{l-1}^{L}\beta_{l0}Z_{\text{lij}}+u_{\text{oj}}\\ u_{\text{oj}}∼N\left(0,\theta^{2}\right) \end{array}$$

i = 1, 2, …, n_j_

j = 1, 2, ...., J

Where:

Y_ij_ is the prevalence of forgoing dental care for individual i in country j. Y_ij_ = 1 if there is non-use of needed care and Y_ij_ = 0 otherwise.

x_ij_ = (x_1ij_, … x_kij_) represents the independent variables for the first level.

z_ij_ = (z_1ij_, … z_lij_) represents the explanatory variables for the second level.

***α***_1_ is a constant (intercept).

***β***_k_ is the k^th^ regression coefficient and measures the *X*_*kij*_unit increase in the log odds ratio.

***β***_l_ is l^th^ regression coefficient and measures the *Z*_*kj*_unit increase in the log odds ratio.

u_j_ is the random effect representing the effect of the j^th^ country.

To measure the proportion of the variance in the prevalence of forgoing dental care that is attributable to differences between countries, we used the intra-class coefficient (ICC) [38,41,42,44,46]. The formula was:

$$\text{ICC}=\frac{\text{Variance}\;2\text{nd}\;\text{level}}{\left(\text{Variance}\;2\text{nd}\;\text{level}+\frac{\pi^{2}}{3}\right)}$$

All statistical analyses were conducted with SAS version 9.2 for Windows (SAS Institute, USA). The multilevel logistic regression models were carried out with the NLMIXED procedure.

## Results

### Prevalence of the non-use of dental care for financial reasons

**Figure 1:**
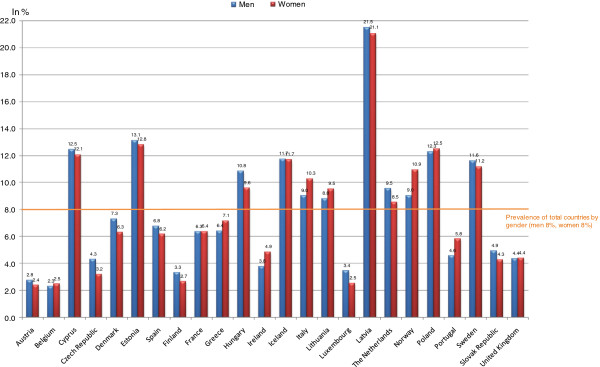
Prevalence of non-use of dental care by country and gender, in 2007.

The prevalence of the non-use of dental care in 2007 ranged from 2.5% (Belgium) to 21.9% (Latvia). For men, it ranged from 2.3% (Belgium) to 21.5% (Latvia), and for women, from 2.4% (Austria) to 21.1% (Latvia; see Figure 1). The prevalence of non-use was above average (8%) for both men and women in 11 of the 24 countries studied, including Italy, the Netherlands, Norway, and Sweden.

**Figure 2:**
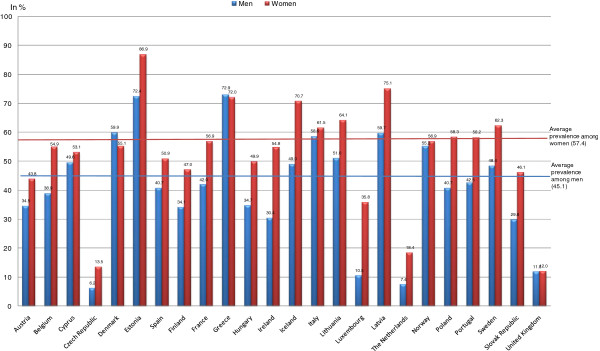
Prevalence of non-use of dental care for financial reasons, among people who renounced care, in 2007.

The prevalence of non-use for financial reasons ranged from 9.6% (Czech Republic) to 80.6% (Estonia; see Figure 2). Specifically, it ranged from 6.2% (Czech Republic) to 72.4% (Estonia) among men and 12.0% (United Kingdom) to 86.9% (Estonia) among women. Thus, in two-thirds of the countries studied, women cited financial reasons as their main reason for not receiving dental care; only in four countries (Czech Republic, 9.6%; the UK, 11.9%; the Netherlands, 12.8%; and Luxembourg, 21.7%) were financial reasons cited by less than one quarter of each sex.

### Socioeconomic inequalities in the non-use of dental care

We found inequalities in the non-use of dental care related to education level when inequality was measured in absolute terms by the difference in rates between the primary school and graduate levels; these ranged from 0.2 (the UK) to 18.1 (Latvia) for men (see Table 3), and 0.1 (Finland) to 19.7 (Latvia) for women (see Table 4). For men, inequalities in non-use were higher in Latvia, Estonia, and Slovakia, while for women, they were higher in Latvia, Norway, and Sweden.

**Table 3:** Prevalence and measures of inequality for non-use dental care, according to the education level among men

	**Prevalence of non-use of dental care (%)**			**Measures of social inequalities in non-use**			
	**Primary education**	**Secondary education**	**Tertiary education**	**Diff (1)**	**RCI (2)**	**RII (3)**	**(95% CI)**
Austria	4.2	2.8	2.2	2.0	−0.076	1.73	0.93--3.23
Belgium	3.7	2.1	1.2	2.5	−0.249	4.27	2.08-8.73
Cyprus	15.2	14.8	7.0	8.2	−0.074	2.22	1.63-3.03
Czech Republic	6.1	4.2	2.7	3.4	−0.099	2.13	1.24-3.67
Denmark	12.2	4.7	3.6	8.6	−0.271	6.78	4.00-11.48
Estonia	22.2	13.6	8.4	13.8	−0.088	3.39	2.57-4.48
Spain	8.2	5.2	4.7	3.5	−0.135	2.63	2.01-3.44
Finland	4.7	3.3	1.5	3.2	−0.192	4.31	2.49-7.45
France	9.4	6.8	4.2	5.2	−0.116	3.03	2.25-4.08
Greece	8.9	4.4	4.2	4.7	−0.183	3.57	2.37-5.38
Hungary	14.5	11.0	7.4	7.1	−0.073	2.21	1.71-2.86
Ireland	5.0	2.3	3.5	1.5	−0.092	1.42	0.81-2.48
Iceland	13.8	9.3	10.8	3.0	−0.096	2.20	1.33-3.63
Italy	10.6	7.9	6.2	4.4	−0.097	2.27	1.89-2.72
Lithuania	15.3	9.4	6.8	8.5	−0.078	2.77	1.86-4.13
Luxembourg	4.1	3.5	2.3	1.8	−0.103	2.67	1.42-5.02
Latvia	32.4	20.3	14.3	18.1	−0.107	3.42	2.66-4.40
The Netherlands	8.9	10.8	9.9	−1.0	−0.067	0.85	0.60-1.20
Norway	13.0	8.5	4.8	8.2	−0.192	3.07	2.04-4.62
Poland	13.4	12.0	11.0	2.4	−0.039	1.21	1.00-1.46
Portugal	5.2	2.6	0.9	4.3	−0.173	8.45	3.10-23.00
Sweden	14.8	12.3	8.6	6.2	−0.092	2.08	1.45-2.97
Slovak Republic	14.6	5.1	1.9	12.7	−0.109	11.5	6.28-20.96
United Kingdom	3.9	4.7	4.1	−0.2	−0.005	1.14	0.73-1.78

Calculated by the authors.

*Abbreviations*: *EU-SILC* European Union Survey Statistic on Income and Living Conditions, *Diff* Difference, *RCI* Relative Concentration Index, *RII* Relative Inequality Index, *CI* Confidence Interval.

Eurostat, EU-SILC 2007.

**Table 4:** Prevalence and measures of inequality for non-use of dental care, according to the education level among women

	**Prevalence for non-use of dental care (%)**			**Measures of social inequalities for non-use**			
	**Primary education**	**Secondary education**	**Tertiary education**	**Diff (1)**	**RCI (2)**	**RII (3)**	**(95% CI)**
Austria	3.2	1.8	2.2	1.0	−0.123	1.93	1.05-3.54
Belgium	4.0	2.1	1.6	2.4	−0.204	4.70	2.36-9.37
Cyprus	15.1	11.2	9.1	6.0	−0.112	2.43	1.71-3.43
Czech Republic	5.9	2.7	2.4	3.5	−0.126	2.86	1.71-4.77
Denmark	8.7	5.3	2.7	6.0	−0.251	3.59	2.01-6.40
Estonia	17.9	15.4	8.5	9.4	−0.132	2.90	2.26-3.73
Spain	7.2	4.5	9.1	−1.9	+0.176	2.44	1.81-3.29
Finland	2.0	2.9	1.9	0.1	−0.115	1.66	0.86-3.19
France	10.3	5.4	4.6	5.7	−0.084	3.09	2.25-4.24
Greece	8.6	5.0	5.2	3.4	−0.178	2.58	1.73-3.85
Hungary	13.9	9.2	6.6	7.3	−0.091	2.64	2.07-3.38
Ireland	4.4	3.4	5.6	−1.2	+0.004	0.64	0.40-1.01
Iceland	16.8	10.4	6.9	9.9	−0.166	3.00	1.70-5.27
Italy	11.6	8.8	9.4	2.4	−0.053	1.86	1.56-2.20
Lithuania	14.5	12.5	7.5	7.0	−0.074	2.91	2.01-4.20
Luxembourg	2.7	3.1	1.7	1.0	+0.000	1.21	0.55-2.61
Latvia	33.7	22.1	14.0	19.7	−0.120	3.01	2.39-3.77
The Netherlands	9.9	7.5	9.9	0.0	+0.050	0.75	0.51-1.10
Norway	18.1	9.3	7.4	10.7	−0.154	3.42	2.31-5.05
Poland	17.7	12.7	11.0	6.7	−0.009	1.62	1.35-1.94
Portugal	5.8	5.0	2.0	3.8	−0.208	2.48	1.30-4.71
Sweden	19.0	11.9	8.3	10.7	−0.103	2.76	1.88-4.06
Slovak Republic	11.5	3.9	2.3	9.2	−0.117	3.97	2.26-6.97
United Kingdom	3.2	4.3	4.7	−1.5	+0.006	0.69	0.44-1.07

Calculated by the authors.

*Abbreviations*: *EU-SILC* European Union Survey Statistic on Income and Living Conditions, *Diff* Difference, *RCI* Relative Concentration Index, *RII* Relative Inequality Index, *CI* Confidence Interval.

Eurostat, EU-SILC 2007.

Using the RCI, we again found, in most countries, inequalities in the rates of non-use between education levels, mainly for men. Negative values, as mentioned above, suggest that the inequality favours the better educated, while positive values suggest that the inequality favours the less educated. The RCI ranged from −0.005 (the UK) to −0.271 (Denmark). For women, the inequalities sometimes favoured the less educated, with concentration indexes ranging from −0.009 (Poland) to 0.176 (Spain). We found mainly negative values for men in all European countries studied, while women had positive values in countries such as Spain, Ireland, the Netherlands, and the UK.

Results for the RII revealed strong social inequalities in the non-use of dental care. For men, the RII ranged from 1.21 (Poland) to 11.50 (Slovakia). The lowest RIIs overall were observed in Poland, Sweden, the Czech Republic, and Ireland, while the highest were Slovakia, Portugal, Denmark, and Finland. For women, the RII ranged from 1.62 (Poland) to 4.70 (Belgium); countries such as Poland, Italy, and Austria had the lowest values, while Belgium, Slovakia, Denmark, and France had the highest values. For Ireland, the Netherlands, and the UK, the confidence interval of the RII indicated no inequality in the non-use of dental care between education levels for both men and women.

### Socioeconomic determinants of the non-use of dental care

The five logistic regression models applied to all European countries considered in this study identified a social gradient in the non-use of dental care related to education level. For example, in Model 3 of Table 5, which includes macroeconomic variables, those with a primary or high school education were more likely not to receive dental care (odds ratios [ORs] = 1.57 and 1.21, respectively) than people with a higher education level.

**Table 5:** Logistic regression of the probability of non-use of dental care in 24 European countries

	**Model 1**		**Model 2**		**Model 3**		**Model 4**		**Model 5**	
	**OR**	**CI 95%**	**OR**	**CI 95%**	**OR**	**CI 95%**	**OR**	**CI 95%**	**OR**	**CI 95%**
**Age**	0.995*	0.994-0.997	0.996*	0.994-0.997	0.997*	0.995-0.998	0.995*	0.994-0.997	0.997*	0.995-0.998
**Sex**										
Men	ref.		ref.		ref.		ref.		ref.	
Women	0.92*	0.89-0.95	0.92*	0.89-0.95	0.92*	0.89-0.95	0.91*	0.88-0.94	0.92*	0.89-0.95
**Education level**										
Tertiary	ref.		ref.		ref.		ref.		ref.	
Primary	1.39*	1.33-1.44	1.37*	1.32-1.43	1.52*	1.45-1.59	1.37*	1.31-1.43	1.51*	1.44-1.58
Secondary	1.23*	1.18-1.28	1.22*	1.18-1.27	1.14*	1.09-1.20	1.21*	1.16-1.26	1.15*	1.10-1.20
**Marital status**										
Married	ref.		ref.		ref.		ref.		ref.	
Divorced/Separate	1.64*	1.57-1.72	1.67*	1.59-1.75	1.61*	1.53-1.70	1.63*	1.54-1.72	1.57*	1.49-1.66
Never married	0.94*	0.90-0.98	0.95*	0.91-0.99	1.04	0.998-1.091	1.00	0.96-1.05	1.03	0.99-1.08
Widow	1.24*	1.17-1.31	1.23*	1.17-1.31	1.13*	1.06-1.20	1.23*	1.15-1.31	1.12*	1.05-1.20
**Standard of living**										
Quartile 4	ref.		ref.		ref.		ref.		ref.	
Quartile 1	1.04*	1.001-1.085	1.04	0.998-1.082	1.01	0.96-1.05	1.03	0.98-1.07	1.01	0.97-1.06
Quartile 2	0.99	0.95-1.03	0.99	0.95-1.03	0.96	0.92-1.01	0.98	0.94-1.02	0.96	0.92-1.01
Quartile 3	1.02	0.98-1.06	1.02	0.98-1.06	1.00	0.96-1.05	1.01	0.97-1.06	1.00	0.96-1.05
**Professional status**										
Employees/Self-employment	ref.		ref.		ref.		ref.		ref.	
Student	0.55*	0.51-0.59	0.55*	0.51-0.59	0.53*	0.49-0.58	0.57*	0.53-0.62	0.54*	0.50-0.58
Retired/pre-retired	0.87*	0.83-0.92	0.87*	0.82-0.91	0.79*	0.74-0.83	0.85*	0.80-0.90	0.79*	0.74-0.84
Other	1.45*	1.39-1.50	1.44*	1.38-1.49	1.45*	1.39-1.51	1.48*	1.42-1.54	1.46*	1.40-1.52
**Density of dentists**										
>7			ref.						ref.	
<6			1.14*	1.10-1.18					0.93*	0.89-0.98
6-7			1.13*	1.09-1.17					0.92*	0.88-0.96
**Human development Index**										
HDI group 3 (HDI > = 0.952)					ref.				ref.	
HDI group 1 (HDI < = 0.874)					1.51*	1.44-1.58			1.74*	1.65-1.83
HDI group 2 (HDI : 0.874 - 0.952)					0.79*	0.75-0.82			0.86*	0.82-0.90
**codcare**										
1							ref.		ref.	
**0**							1.15*	1.12-1.19	1.32*	1.26-1.37
R^2^	0.0161		0.0167		0.0286		0.0165		0.0312	

Calculated by the authors.

(*)Significant to the error threshold of 5%;

*Abbreviations*: *EU-SILC* European Union Survey Statistic on Income and Living Conditions, *OR* Odds Ratio, *CI* Confidence Interval.

Eurostat, EU-SILC 2007.

However, we found no statistically significant relationships between standard of living (as measured by the quartile of disposable income) and the non-use of dental care. Furthermore, professional activity was associated with the non-use of dental care: employees or self-employed people tended to forgo care more than students (OR = 0.51, 95% confidence interval [CI]: 0.47–0.55) and the retired/pre-retired (OR = 0.79, 95% CI: 0.75–0.83; Model 3). Finally, demographic factors (age, sex, and marital status) were associated with the risk of non-use of dental care. Men went without dental care more often than women, while those who were divorced or separated went without dental care more often than married people (OR = 1.64, 95% CI: 1.57–1.72 in Model 1, and OR = 1.59, 95% CI: 1.52–1.67 in Table 5, Model 3).

Finally, the results of the macroeconomic variables (HDI, density of dentists, and dental care insurance coverage) were also found to have significant relationships with the non-use of dental care. The non-use of dental care was twice as high among people living in low-HDI countries (i.e. HDI ≤ 0.874) compared to those living in high-HDI countries (i.e. HDI ≥ 0.952). Furthermore, dentist density was associated with the risk of non-use of dental care. For example, the risk of non-use of dental care was higher among people living in countries with a dentist density less than 6 (OR = 1.14, 95% CI: 1.10–1.18) or equal to 6–7 (OR = 1.13, 95% CI: 1.09–1.17) compared to those who live in countries where the density exceeds 7 (see Table 5, Model 2). However, after adjusting for HDI, the odds ratio for non-use changed for people who live in countries where dentist density is less than 6 (see Table 5, Model 3). People living in countries with low dental coverage (≤50%) were significantly more likely to report unmet dental needs than those in countries with higher coverage (>50%).

The results of the multilevel analysis (see Table 6) showed the existence of variation across countries in the prevalence of the non-use of needed dental care. The empty model established the variation across countries in the prevalence of non-use of dental care as well as the five other models. Random level 2 ranged from 1.459 in the empty model to 1.178 in the full model. All the individual-level characteristics had significant effects, except for standard of living. Only the coefficient of the fixed effects of the HDI was statistically significant among the contextual-level characteristics. People living in countries with a low HDI were two times more likely to forgo dental care compared to those in countries with a higher HDI (OR = 2.00, CI: 1, 10 to 3.67) (see Table 6, Model 3). In Model 5, none of the contextual explanatory variables was significantly associated with the likelihood of the non-use of dental care.

**Table 6:** Multilevel logistic regression of the probability of non-use of dental care by individual and countries characteristics

	**Model 0**	**Model 1**		**Model 2**		**Model 3**		**Model 4**		**Model 5**	
	**(Empty model)**	**OR**	**CI 95%**	**OR**	**CI 95%**	**OR**	**CI 95%**	**OR**	**CI 95%**	**OR**	**CI 95%**
**Fixed effects**											
Intercept	0.07*	0.07*	0.05-0.09	0.07*	0.04-0.10	0.06*	0.04-0.10	0.08*	0.06-0.11	0.10*	0.06-0.15
**Age**		0.93*	0.90-0.97	0.93*	0.90-0.97	0.93*	0.90-0.97	0.92*	0.89-0.96	0.92*	0.89-0.96
**Sex**											
Men		ref.		ref.		ref.		ref.		ref.	
women		0.997*	0.995-0.998	0.997*	0.995-0.998	0.997*	0.995-0.998	0.995*	0.995-0.997	0.995*	0.99-0.997
**Education level**											
Tertiary		ref.		ref.		ref.		ref.		ref.	
Primary		1.49*	1.42-1.56	1.49*	1.42-1.56	1.49*	1.42-1.56	1.46*	1.38-1.54	1.46*	1.38-1.54
Secondary		1.24*	1.19-1.30	1.24*	1.19-1.30	1.24*	1.19-1.30	1.21*	1.15-1.27	1.21*	1.15-1.27
**Marital status**											
Married		ref.		ref.		ref.		ref.		ref.	
Never married		0.97	0.92-1.01	0.97	0.92-1.01	0.97	0.92-1.01	0.98	0.93-1.03	0.98	0.93-1.03
Divorced/Separate		1.69*	1.60-1.78	1.69*	1.60-1.78	1.68*	1.59-1.78	1.73*	1.63-1.85	1.73*	1.63-1.85
Widow		1.13*	1.06-1.20	1.13*	1.06-1.20	1.13*	1.06-1.20	1.13*	1.05-1.21	1.13*	1.05-1.21
**Standard of living**											
Quartile 4		ref.		ref.		ref.		ref.		ref.	
Quartile 1		1.01	0.97-1.06	1.01	0.97-1.06	1.01	0.97-1.06	1.02	0.98-1.08	1.02	0.98-1.08
Quartile 2		0.97	0.93-1.02	0.97	0.93-1.02	0.97	0.93-1.02	0.98	0.93-1.03	0.98	0.93-1.03
Quartile 3		0.99	0.95-1.04	0.99	0.95-1.04	0.99	0.95-1.04	0.99	0.94-1.04	0.99	0.94-1.04
**Professional status**											
Employees/Self-employment		ref.		ref.		ref.		ref.		ref.	
Retired/pre-retired		0.85*	0.80-0.90	0.85*	0.80-0.90	0.84*	0.80-0.89	0.82*	0.77-0.87	0.82*	0.77-0.87
Student		0.42*	0.39-0.46	0.42*	0.39-0.46	0.42*	0.39-0.46	0.44*	0.40-0.48	0.44*	0.40-0.48
Other		1.42*	1.36-1.45	1.42*	1.36-1.45	1.42*	1.36-1.48	1.41*	1.34-1.47	1.41*	1.34-1.47
**Density of dentists**											
>7				ref.						ref.	
<6				1.06	0.55-2.05					0.90	0.53-1.50
6-7				1.12	0.60-2.11					0.78	0.46-1.33
**Human development Index**											
HDI group 3 (HDI > = 0.952)						ref.				ref.	
HDI group 1 (HDI < = 0.874)						2.00*	1.10-3.67			1.59	0.85-2.97
HDI group 2 (HDI: 0.874 - 0.952)						0.78	0.46-1.32			0.63	0.39-1.00
**Codecare**											
1								ref.		ref.	
0								0.75	0.45-1.25	0.76	0.47-1.23
**Random effects**											
Level 2 : between country variation	1.46*	1.50*	1.18-1.92	1.48*	1.18-1.88	1.31*	1.11-1.54	1.32*	1.09-1.59	1.18*	1.05-1.32
Intraclass coefficient (ICC)	10.28%	11.01%		10.72%		7.61%		7.77%		4.75%	
−2 log likelihood	176679	129187		129187		129178		190685		109675	
Number of countries	24	24		24		24		20		20	

Calculated by the authors.

(*)Significant to the error threshold of 5%

*Abbreviations*: *EU-SILC* European Union Survey Statistic on Income and Living Conditions, *OR* Odds Ratio, *CI* Confidence Interval.

Eurostat, EU-SILC 2007.

ICCs, which measure the proportion of the variance in the prevalence of forgoing dental care that is attributable to differences between countries, decreased from 10.28% in the empty model (i.e. the model without the individual and contextual variables) to 4.75% in the full model (Model 5). Thus, according to these ICCs, 10.28% of the variation in the prevalence of the non-use of dental care was due to unmeasured characteristics associated with the respondents’ countries. A small intra-class coefficient suggests much greater heterogeneity within than between countries.

## Discussion

In this study, we examined the socioeconomic determinants of the non-use of dental care across 24 European countries. Our results showed that socioeconomic inequalities are present in the non-use of dental care in Europe, but their magnitude depends on the method of measuring inequality.

The varying measures of inequality used in this study showed that the non-use of dental care was associated with education level in Europe. Generally, the non-use of dental care was highest among people with the lowest level of education. However, we observed differences in this for men in the Netherlands and women in Spain and the UK. We noted that the use of absolute differences as a measure of social inequality seemed insufficient, as they could not take into account the results for intermediate levels of education. However, other measures of inequality (RCI and RII) presented similar results and confirmed those of other studies on the utilization of health care services [3,6,15]. Furthermore, comparisons of the confidence-interval values for RII show that the effect of socioeconomic inequality on the non-use of dental care is strongest in Denmark. In Italy, Sweden, Poland, Hungary, Czech Republic, and Cyprus, the effect of socioeconomic inequality on the non-use is strong for men. For women, the RII is strongest in Latvia and less important in Poland and Italy.

Access to dental care was unevenly distributed across educational levels. Several previous studies have shown that a socioeconomic distribution favouring the richest people influences the probability of visiting a dentist in all OECD countries. For example, people with higher incomes are more likely to have received dental care in the last 12 months than those with lower incomes [3,6,10,12,15]. Furthermore, we found that inequalities in the non-use of dental care were not consistently lower in Scandinavian countries, despite their reputation for egalitarian political and social structures [31]: although the rates of non-use were low in general, access to dental care was still limited for disadvantaged people with a lower education level. Indeed, among the various barriers that may curb the utilization of dental care, financial problems were reported as the main reason in half of the countries studied, and this increased to two-thirds of the countries if we considered only women. This is mainly observed in countries where people’s direct share of the cost of dental care is more than 50% of total dental expenses, such as Denmark (70.5%), Norway (75.4%), Iceland (80.6%), and Poland (63.9%) [12]. Thus, it is not surprising that the rates of the non-use of dental care are higher in these countries. Furthermore, financial reasons (as cited by respondents) included various aspects not recorded in the EU-SILC survey that relate to the organisation and financing of healthcare in different countries, such as the extent of coverage under social-security schemes, eligibility criteria, care-support criteria, or policies that require higher patient co-payments [14]. However, even in countries that provide universal dental coverage (Austria, Spain, Poland [23], Denmark, Finland, Italy, and the United Kingdom [47]), unequal access to dental care remains common. Paradoxically, our results show that financial barriers were the primary reason cited in most of these countries. In Canada, a study on the relationship between dental insurance and the use of dental services [48] showed that the probability of visiting a dentist was lower among people with regular income and with low education levels even when they had insurance, compared with those with high income and high education levels. Other researchers [49] have indicated that ‘having dental coverage helps, but access and utilization problems remain even for those have it’.

Some notable findings from this study set it apart from previous research in the field. First, the risk of non-use of dental care was not associated with lifetime or personal income, although the association between the probability of visiting the dentist and income has been established [3,10,12,50]. This result can be explained by the fact that the choice to seek care is not simply a financial one; the type of dental care needed and provider availability would also factor into the decision. As observed in Canada, universal health insurance programs can help limit the influence of income on access to dental care [13].

Second, our results showed a statistically significant relationship between the population density of dentists and the probability of the non-use of dental care. Researchers have not observed this in other analyses, such as the association between the probability of the use of generalists and specialists and the density of medical professionals [51]. Dentist density plays an important role in the development of geographical inequalities in the use of health services. Indeed, the likelihood of going without dental care is higher among people living in a country with a low density of dentists (see Model 2, Table 5). In France, a study of people aged 60 years and over living at home [52] found that the density of dentists was a significant factor in their access to dental services. In Sweden, the lack of access to dental care services explained about 60% of the socioeconomic differences in poor oral health among men and women, while behavioural factors (or lifestyles) explained only 29% [36].

Our use of the HDI in this analysis of inequalities in the non-use of dental care expands the existing literature on the importance of this index. In future research, the HDI could be used to highlight the relationship between a country’s socioeconomic development and the extent of inequalities in the non-use of dental care. The HDI has the advantage of combining three important indicators of social well-being: GDP per capita, school enrolment, and life expectancy at birth. Thus, it is reasonable to propose that the risk of the non-use of dental care would be higher among people living in countries with low HDIs than among those living in countries with high HDIs. In the present study, most Eastern European countries had low HDI, and therefore, higher risk of non-use. Our results are consistent with those of the existing literature that used measures such as GDP to index a country’s development [8]. Indeed, previous researchers have revealed socioeconomic inequalities in the use of dental services that favour the richest people in most countries, and greater levels of relative inequality in countries with very low income than those with very high income.

By comparing the results presented in Tables 5 and 6, it is clear that the standard logistic regression models (at level 1) tend to provide regression coefficients that are close to those obtained with multilevel logistic regression models, particularly concerning individual characteristics. However, the results differed for the contextual variables, with the exception of the HDI. Despite these differences, the two types of regression analysis produced results with the same directionality, namely that the probability of the non-use of dental care varies depending on the characteristics of the country. Multilevel analysis provided more robustness for estimating the effects of contextual variables on the probability of the non-use of needed dental care. Thus, all things being equal, the density of dentists and the level of insurance coverage for basic dental care were no longer statistically significant in the non-utilization of dental fixed effects. Our results show only a small change in the probability of the non-use of dental care across European countries, which is due to unobserved contextual characteristics. Thus, these results suggest that the country itself had a small impact on the prevalence of the non-use of dental care.

Because our study is based on data collected from 24 European countries that implemented the same data collection tools and the same methodology, we were able to easily compare the results across countries. However, in practice, there may have been many differences in survey administration across the various countries, and this may have introduced some biases. For example, data on the density of dentists could be questionable due to differing data collection dates. Nonetheless, these data can still be considered one aspect of each country’s healthcare system that is likely to influence access to care. Another limitation to the current study is the absence of data relating to individuals’ enrolment in health insurance programs that cover dental care, although previous studies have shown that inequalities in access were still strong in countries with full dental-care coverage under their universal health care systems [23,47]. As our data derived from the EU-SILC, our study encompassed a much wider range of countries compared with other recent research [8,15]. This allowed us to offer a much broader view of the inequalities and socioeconomic determinants of the non-use of dental care in Europe.

Moreover, our outcome variable was defined using the EU-SILC concept of unmet needs. Indeed, the need for unmet care may be defined as an individual’s perceived need (subjective need), a need diagnosed by a professional (objective need), or both. People may well perceive the need to see a dentist and seek care within a 12-month period, but then be required to forgo some care for various reasons during the same period. In fact, the prevalence of unmet dental need as calculated according to the EU-SILC data represents a combination of these two types of need in some respects.

## Conclusion

We found that socioeconomic inequalities in the non-use of dental care are present in every country in Europe, but the magnitude of these inequalities depends on the measure used. Controlling for age, sex, employment status, density of dentists, and marital status, education level appears to be a much stronger determinant of inequalities in the non-use of dental care than disposable income, for which our results showed almost no significant associations. Thus, education level seems to play an important role in the non-use of dental care among European citizens; professional status also appeared as an influential factor. Of course, the observed differences may be explained by other individual socioeconomic determinants and various environmental characteristics not included in this study.

Multilevel analysis illustrated the variability between countries in the prevalence of forgoing dental care. However, this variation appears to be due to unmeasured characteristics of the countries. Overall, our study shows that socioeconomic inequalities in the non-use of dental care exist at both the individual (intra-country) and collective (inter-country) levels. Therefore, to be the most effective, policies to combat social inequality in Europe should address both of these levels.

## Competing interests

The authors declare that they have no competing interests.

## Authors’ contributions

AT was the principal investigator of this study; he conducted the statistical analysis and wrote the manuscript. NL conducted the statistical analysis and wrote the manuscript. All authors read and approved the final manuscript.
